# Talking about risk in the context of genomic tests (TARGET): development and evaluation of an educational program for clinicians

**DOI:** 10.1007/s10549-019-05316-7

**Published:** 2019-06-14

**Authors:** L. Fallowfield, I. Solis-Trapala, R. Starkings, S. Catt, S. May, V. Jenkins

**Affiliations:** 1 Sussex Health Outcomes Research & Education in Cancer (SHORE-C), Brighton & Sussex Medical School, University of Sussex, Falmer, BN1 9RX UK; 2 Faculty of Medicine and Health Sciences, Keele University, Staffordshire, ST5 5BG UK

**Keywords:** Risk of recurrence, Genomic test results, Breast cancer, Chemotherapy, Communication

## Abstract

**Purpose:**

Gene expression profiling (GEP) test scores calculate risks of recurrence and likely benefit of adjuvant chemotherapy in ER-positive, HER2-negative, early-stage breast cancer. As health literacy and numeracy skills in the general population are poor, healthcare professionals (HCPs) require a wide repertoire of communication skills to explain clearly risk of recurrence scores (RSs) and uncertainty. We developed and evaluated an educational program for HCPs discussing GEP test results and adjuvant treatment.

**Methods:**

Eight-hour workshops contained elements aimed at improving knowledge, communication skills and self-awareness; these included the science underpinning GEP tests, an interactive risk psychology lecture, exercises and facilitated group discussions regarding seven filmed scenarios involving discussions about high, intermediate and low RSs. Attendees were recorded explaining RSs with patient simulators pre and post workshop. Researchers, blinded to time point, analysed recordings using a study-specific scoring system. Primary objective outcomes were improvements post workshop in HCPs’ competence and confidence when communicating 17 pre-specified key information areas. We estimated odds ratios (OR) using conditional logistic regression to compare pre- and post-workshop scores.

**Results:**

65 HCPs attended. Objective analyses revealed significant positive shifts post workshop which included explaining GEP tests (OR 2.98; 95% CI 1.38–6.42; *P* = .001), recurrence RSs (OR 3.99; 95% CI 1.72–9.25; *P* < .001), benefits of chemotherapy (OR 3.99; 95% CI 1.82–8.75; *P* < .001; and harms OR 2.31; 95% CI 1.37–3.92; *P* < .001) using jargon free language (OR 5.29; 95% CI 2.27–12.35; *P* < .001). Patient simulator assessments also showed significant improvements as did HCPs’ self-assessments and ratings of their self-confidence when discussing different GEP tests with diverse patient types (*P* < .001).

**Conclusion:**

These short, intensive, interactive TARGET workshops significantly improved HCPs’ communication about GEP results in ways likely to promote more informed decision-making by patients about chemotherapy.

**Electronic supplementary material:**

The online version of this article (10.1007/s10549-019-05316-7) contains supplementary material, which is available to authorised users.

## Introduction

There have been exciting advances in diagnostics, surgical and radiotherapy techniques and systemic therapies for women with early-stage breast cancer (EBC). Better understanding about the molecular biology of cancer has permitted more targeted treatment, and thus many patients have genuine prospects of cure or living longer. Most patients with cancer want collaborative roles in treatment decision-making [1], but discussions about the logic and rationale behind treatment recommendations have become increasingly complex and demand excellent communication skills. Decision-making about adjuvant chemotherapy in particular requires careful balancing between likely absolute benefits in terms of preventing recurrence versus the unpleasant side effects and inconvenience of treatment. Explaining risks and benefits to patients is never easy and health literacy and numeracy skills are frequently poor in the general populations of the US, Canada, Australia and EU [2, 3]. Perceptions about health risk information are strongly related to numeracy so women with inadequate numeracy skills are more likely to overestimate their chances of dying from breast cancer and the absolute risk reduction of breast cancer screening [4, 5]. Many patients also believe that in the context of life-threatening disease, more treatment is better than less.

Various decision aids such as Adjuvant! On-line were designed to help clinicians determine and discuss treatment recommendations. Studies show, however, that patients’ numeracy profoundly affects their ability to interpret numerical estimates of treatment efficacy [6]. In the UK, NHS Predict V2.1 is a prognostication and treatment benefit tool providing survival estimates 5 and 10 years following surgery with and without adjuvant therapy (hormone therapy, chemotherapy and trastuzumab) [7]. Clinicians can show patients relevant information with text, graphs, charts or in icon arrays. Clinico-pathologic features of the tumour are good prognostic indicators for most patients, but for some, the additional assessment of the risk of recurrence, not just survival estimates, is also pertinent for decision-making. Linking such decision-aid information to other factors that might determine treatment recommendations can be confusing for patients.

Several gene expression profiling (GEP) tests including EndoPredict^®^ (EPclin score), Onco*type* DX^®^ Breast Recurrence Score and Prosigna^®^ are approved to guide adjuvant chemotherapy decisions for patients with oestrogen receptor (ER)-positive, human epidermal growth factor receptor 2 (HER2)-negative and lymph node (LN)-negative breast cancer. Discussing the classifications of high, intermediate or low risk are somewhat illusory as all GEP recurrence RSs are on a continuum with their cut-offs based on clinical trial data. In one survey, a third of women receiving risk of recurrence test results did not fully understand these discussions [8]. Health literacy may affect patients’ retention of RS test results, their capacity for processing information, understanding and decision-making [5]. Results from a study examining patients’ understanding and preferences for six different Oncotype DX risk score formats of increasing complexity showed high error rates irrespective of health literacy or numeracy [9].

Even when RSs suggest high or low risk of recurrence, interpretation of these is subject to various unconscious biases. The personality characteristics of patients and doctors, especially their tolerance of uncertainty, may contribute to indecision or seemingly irrational choices [10]. Clinicians less tolerant of uncertainty may, when encountering ambiguous clinical situations, order more diagnostic tests or extra treatments than colleagues who are more accepting [11]. Furthermore, they may be less likely to discuss uncertainty openly with patients or engage in shared decision-making [12, 13]. Anxious patients with lower tolerances of ambiguity are often uncomfortable forgoing all possible treatments including chemotherapy even if benefits are modest or negligible.

Consequently health care professionals (HCPs) require personal numeracy expertise and wide repertoires of communication skills when explaining concepts to patients craving certainty about their prognoses and treatment outcomes.

As clinicians admit to communication difficulties when discussing GEP test scores, we developed an 8-h educational program—Talking About Risk in the context of GEnomic Tests (TARGET). The intervention’s central aim was to assist HCPs when communicating Onco*type*DX^®^ and EndoPredict^®^ results thus helping their patients to receive clearer information about putative adjuvant chemotherapy benefits permitting more educated shared decision-making.

## Workshop development and contents

Research shows that improving both the competence and the self-confidence of HCPs are necessary prerequisites for the effectiveness of educational initiatives and transfer of skills into a clinic setting. Programs must include areas that enhance skills development (SD), knowledge acquisition (KA) and personal awareness (PA) [14–16].

We developed training materials following review of the risk literature and discussions with key clinicians and scientists. We mapped out the difficulties encountered explaining high, intermediate and low RSs together with the added challenges faced when communicating with patients with diverse personality and socio-educational characteristics. Other difficulties included developing understandable explanations that survival following adjuvant treatments is dependent on the risk of distant recurrence of breast cancer, how to transform percentages into different graphical representations, translating risks of recurrence into simple but non-patronising language, and the pros and cons of using analogies to describe risk.

We rehearsed patient simulators (actors) experienced in improvisation to create different characters and filmed their unscripted GEP test result consultations with cancer clinicians. This methodology had proved successful in our educational initiatives improving communication about clinical trials [17, 18]. TARGET workshop components and their educational aims are shown in Table 1.

**Table 1:** TARGET contents and educational aims

	Content	Knowledge acquisition	Skills development	Personal awareness
Introduction	An interactive session with a lecture about the psychology of risk and uncertainty and group exercises on basic numeracy, tolerance of uncertainty and frequency descriptors of numerical concepts	*√*		*√*
Module 1	An interview with a basic scientist about the principles underpinning GEP tests	*√*		*√*
Module 2	2 scenarios in which 2 oncologists discuss a high and a low risk, EndoPredict^®^ test results with patients	*√*	*√*	*√*
Module 3	3 scenarios in which different oncologists and a specialist nurse discuss low, intermediate and high Onco*type*DX^®^ RSs	*√*	*√*	*√*
Module 4	2 further Onco*type*DX^®^ scenarios filmed following the publication of the TAILORx trial which changed some of the cut-offs and classifications of risk	*√*	*√*	*√*

Workshops lasted approximately 8 h and were facilitator-led encouraging group discussion between participants about the specific communication skills displayed by oncologists in different scenarios, and practical exercises on different means of conveying risk information needed for decision-making and handling varied patient reactions. Workshops concluded with attendees generating lists of optimal and necessary points that must be covered when discussing GEP test results.

After designing, piloting and refining the program, we conducted an evaluation of its efficacy and acceptability with HCPs actively engaged in discussing RSs.

## Methods

### Participants

Participants based in the UK were recruited through SHORE-C website adverts and flyers distributed at breast cancer meetings following presentations showcasing filmed materials. Workshops were accredited 9 Continuing Professional Development (CPD) points from the Royal College of Physicians. Brighton & Sussex Medical School Regional Ethics Committee approved and sponsored the study (ref: ER/RMLS21/1) funded by the Breast Cancer Research Foundation.

### Assessments

On the morning prior to and the afternoon following the workshop, participants were recorded explaining RSs with simulated patients. Their case studies were based on real clinical situations, and included consultations with low-risk patients, who nevertheless wished to have chemotherapy, and higher risk patients averse to chemotherapy. Before recording, participants read their assessment case study, the GEP test result, and any associated reports, printed handouts or additional information which they normally used in their own clinical practice, such as Predict outputs. They could access computer notes and displays if required. All simulated patients were experienced in improvisation and well briefed about breast cancer, its symptoms and reasons for the consultation. To enhance authenticity, different actors and scenarios were used pre and post workshop.

Participants gave written consent to all assessments prior to the first recording.

### Assessments of recorded interviews

There were three separate assessments of all digitally recorded interviews: (1) objective assessment by trained researchers, (2) self-assessment by attendees and (3) patient simulator assessment.

An independent data manager assigned random numbers to each digital interview which researchers assessed using a study-specific checklist, blinded to assessment time point. Researchers’ coding involved checking the presence of 17 key communication and information areas, together with the HCP’s competence (‘did not do this’, ‘not very well’, ‘reasonably well’, ‘very well’). Areas covered issues such as explaining the patient’s risk of recurrence with and without treatment, implications for survival, making appropriate use of print outs/graphs to aid understanding about RSs and other general communication behaviours including structuring and summarising information.

Following each recorded interview, HCPs self-rated their performances on all 17 areas, and how satisfied and confident they felt with their interviews and chemotherapy decisions.

Post interview, simulated patients also scored their HCP’s communication skills on the same 17 items, indicated the decision made and provided general comments for feedback.

### Self-confidence questionnaires

Instruments similar to those used in previous assessments of our educational interventions [17, 18] were adapted for attendees to self-rate their confidence pre and post workshop when discussing 9 general aspects of RSs on a scale from 0 (none) to 10 (very confident).

### Self-awareness, numeracy and communication exercises

During the interactive lecture on risk and uncertainty, attendees completed three exercises: (1) measurement of their own predisposition towards uncertainty, (2) a basic numeracy test and (3) an exercise about verbal descriptors of frequencies.

1. The Intolerance of Uncertainty Scale (IUS) measures responses to uncertainty, ambiguous situations and the future [19]. Intolerance of uncertainty is the tendency to consider possibilities of a negative event as unacceptable and threatening irrespective of the probability of its occurrence. Individuals with high intolerances attempt to make seemingly less risky choices, so the scale has utility when examining HCPs’ decision-making behaviours.
2. Basic numeracy-related skills, in particular the facility to transform probabilities into proportions, proportions into percentages and vice versa, are vital when helping patients to understand risk. Participants completed a basic numeracy assessment (based on Schwartz) probing their numeracy abilities [20].
3. Attendees also completed a short exercise examining frequencies and verbal descriptors. They imagined a fictitious drug for indigestion and indicated how many people out of 100 might get various side effects described as ‘fairly common’, ‘often’, ‘unlikely’ and ‘very rare’.

At the workshop conclusion, attendees rated the quality of the educational materials, specific aspects of the content, and whether or not they would recommend the program to colleagues.

### Hypotheses

Our a priori hypotheses were that post workshop (a) attendees’ communication when discussing GEP test results would improve, namely that their competence would be measurably better, and (b) that they would feel more confident conducting these interviews, that is, their self-efficacy/self-confidence would be enhanced.

### Statistical analyses

As the primary objective outcome was the analysis of participants’ digitally recorded interviews with simulated patients, pre and post workshop, each of the two researchers coding interviews performed rate–rerate reliability checks on their own assessments and inter-coder reliability checks for 10% of each other’s interviews. Both rate–rerate and inter-coder reliability were examined using Kappa coefficients. A conditional logistic regression model was used to compare pre- and post-workshop scores for confidence levels and for the presence and competent handling of each key communication item, using a numeric score for responses 0 (did not do this), 1 (not very well), 2 (reasonably well), 3 (very well). This model estimates, for each individual, the probability that a score is observed post workshop rather than before it. This probability is expressed in terms of the difference between pre- and post-scores. The parameter of interest in the model is the odds ratio (OR). The larger the OR, the more likely it is that higher scores are observed post workshop. No distributional assumptions are required for the scores. Thus, this approach provides a robust method for before-and-after comparisons. The key data used by the estimation procedure are numbers of attendees with different scores at the two time points. Large positive ORs occur when changes fall towards larger score values rather than smaller values. Each item reflects a distinctive communication area, the interpretation of which is of interest on its own. We do not make an overall communication recommendation based on amalgamation of all items; neither do we control the overall error rate. Consequently, corrections for multiple testing are not necessary. The results are exploratory and all inferential statements are valid marginally; that is, they are to be interpreted individually for each item [21, 22].

We also analysed the levels of agreement between (a) coder and HCP, (b) coder and actor and (c) actor and HCP on their assessment of competence for every information area post workshop. For each of the communication areas, firstly the rating was dichotomised as (0 = ‘did not do this’ or ‘not very well’ and 1=‘reasonably well’ or ‘very well’), and secondly a binary variable was formulated to indicate agreement (1 = Yes, 0 = No) between each pair of raters for each recording. We used a logistic regression model for this variable to estimate an overall odds of agreement in each information area considered. To yield chance-corrected measures of agreement, models incorporated corrections for chance agreement in the linear predictor following Lipsitz et al. [23]. The total score for Intolerance of Uncertainty Scale was analysed using a linear regression model with attendees’ levels of confidence on each area of information as explanatory variables.

## Results

Between March and July 2018, seven workshops were attended by 65 breast cancer specialists (38 women; 27 men), of whom 32 were oncologists, 24 surgeons and 9 nurses.

### Analyses of digital recordings

The inter-rater (two coders) and rate–rerate reliability showed good agreement (*k *= .9123; SE = .0712; *k *= .8101, SE = .1517 , respectively). A descriptive analysis of pre- and post-workshop scores (supplementary Tables D–G) show the trends of improvement in most communication areas and HCP self-reported confidence overall by speciality.

**Fig. 1:**
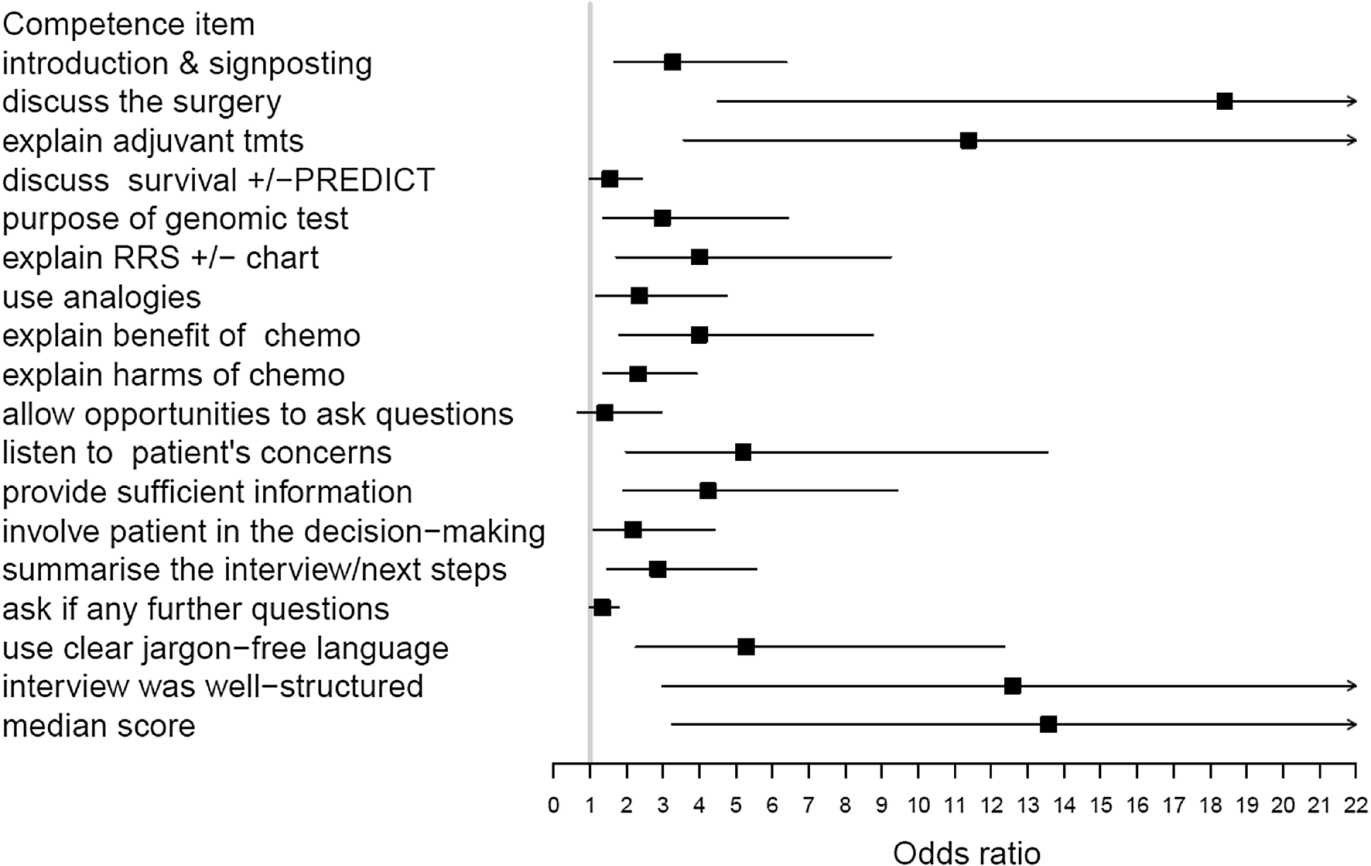
Objective analysis of recordings. Forest plot shows odds ratios (95% confidence intervals) of improved scores including overall median score

Participants’ objectively assessed competence when communicating with patients about GEP test scores improved significantly post workshop (Fig. 1 and Supplementary Table A). Significant changes were recorded by coders for the majority of key information areas and in structuring discussions. There was also a positive overall significant median change.

**Fig. 2:**
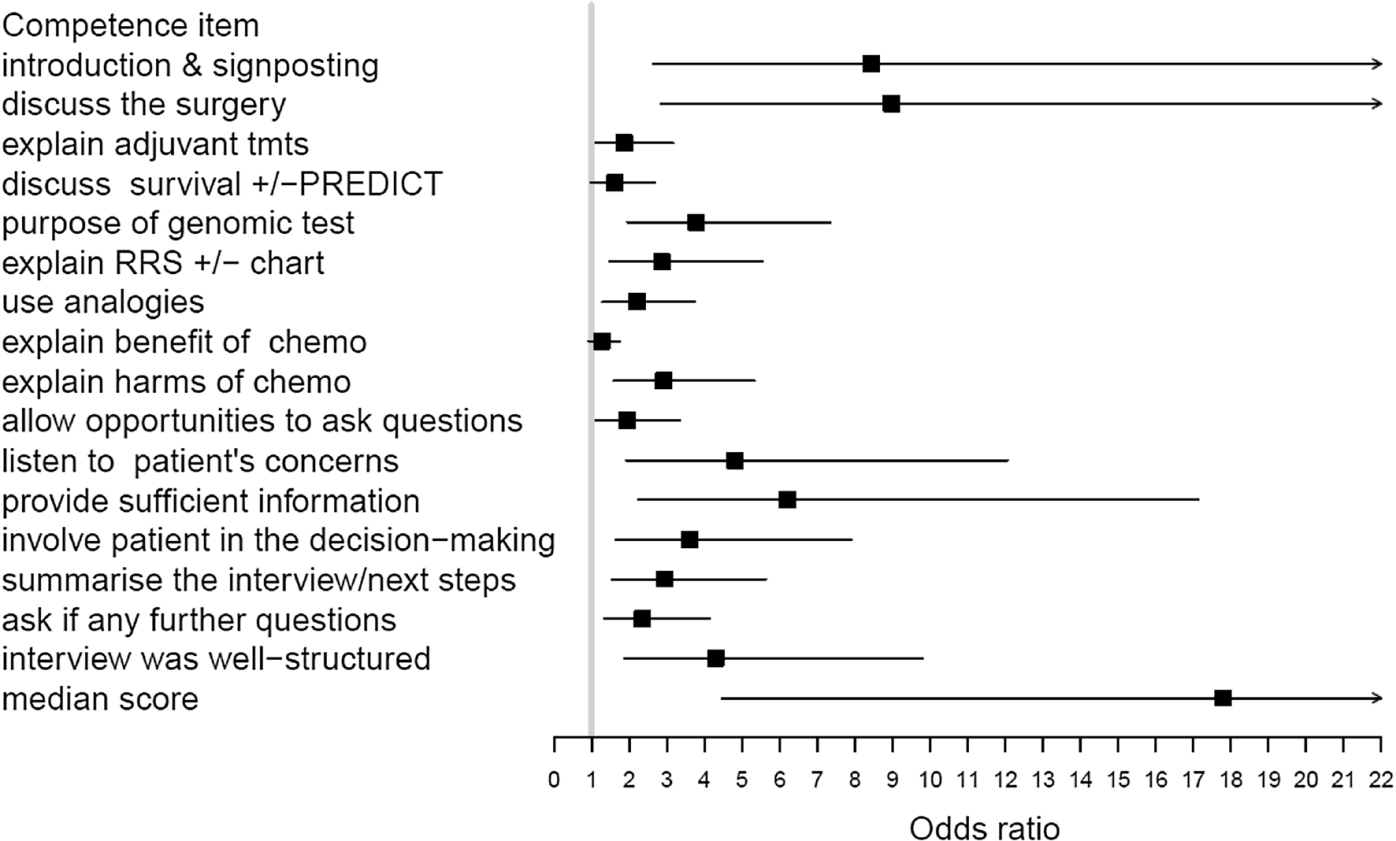
Odds ratios (95% confidence intervals) of improved scores for simulated patients’ assessment of communication behaviours including overall median score

**Fig. 3:**
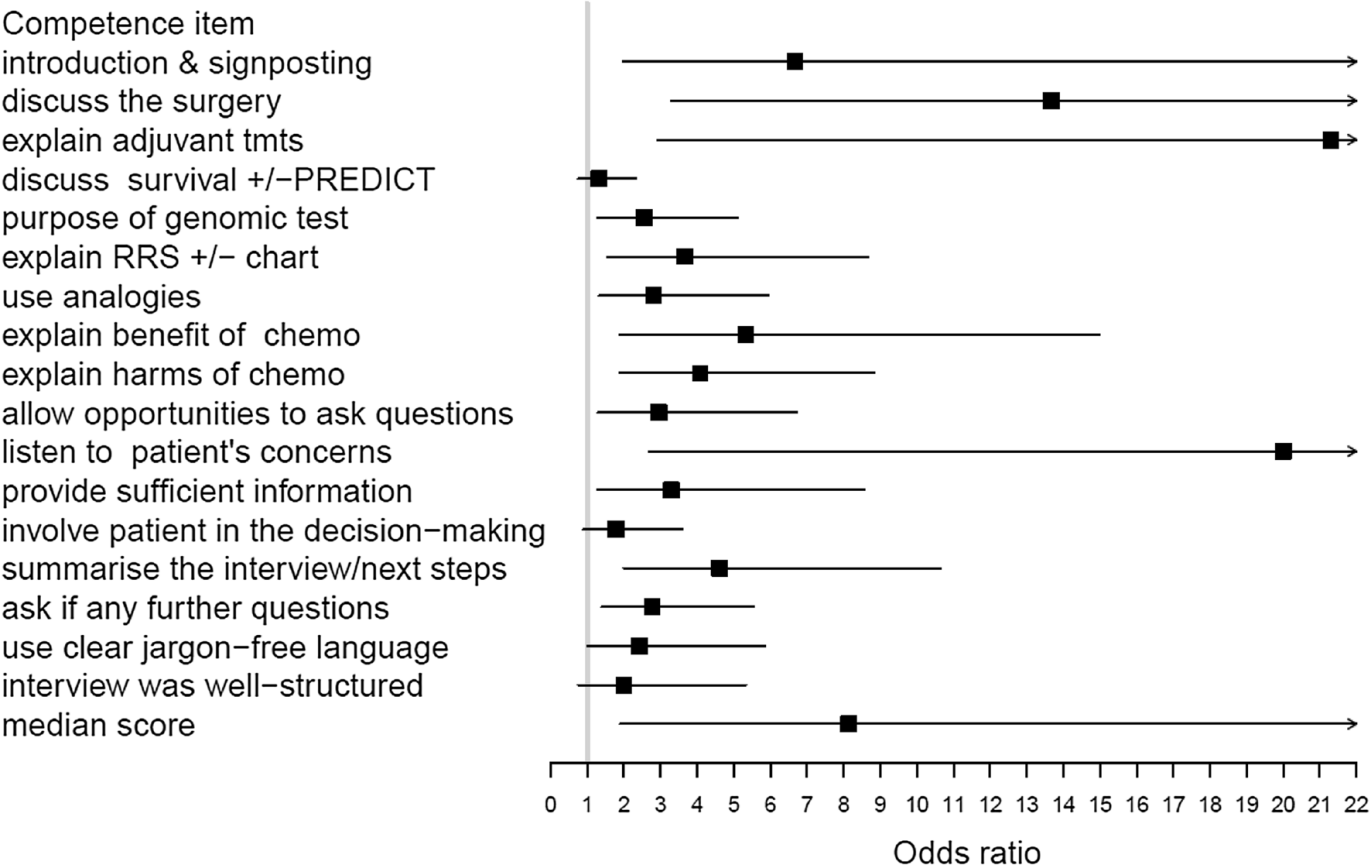
Odds ratios (95% confidence intervals) of improved scores for HCPs’ assessment of communication behaviours including overall median score

The odds ratios for the simulated patients’ ratings of attendees’ 17 key communication behaviours (Fig. 2 and Supplementary Table B) show many positive changes including structuring interviews, when explaining the purpose of GEP tests and the risk of recurrence results. Similarly participants’ self-ratings of their communication skills were significantly higher post workshop (Fig. 3 and Supplementary Table C).

High levels of agreement (concordance) were observed between coders, HCPs and simulated patients for ratings of “reasonably well” and “very well” responses across the 17 items. There were too few disagreements in these data for some items to make any further analyses of agreement worthwhile.

Attendees expressed significantly higher levels of confidence with their discussions following post-workshop interviews (OR 3.27; 95% CI 1.53–6.99; *P* = .0022).

### Intolerance of Uncertainty Scale (IUS)

Participants’ mean IUS score was in the normal range but 12/65 (18%) had high scores, 7 of whom were extremely intolerant of uncertainty (Supplementary table H). Higher intolerance of uncertainty was associated with lower self-rated confidence when discussing prognosis with patients with EBC and when discussing intermediate RSs (beta = − 1.67; 95% CI − 2.89 to − .45; *P *= .009; beta = − 1.76; 95% CI − 3.15 to − 0.37, *P *= .016). Higher intolerance of uncertainty scores was also associated with higher levels of self-rated confidence when discussing high RSs generally and when communicating with patients of lower socio-educational background (beta = 1.81; 95% CI 0.16–3.45; *P *= .035; beta = 1.95; 95% CI 0.18–3.72, *P *= .035.)

### Basic numeracy

Correct responses to the basic numeracy items are shown in Table 2. The poorest performance (only 58% correct) was for question 3—calculating the expected frequency of an event based on its probability of occurrence.

**Table 2:** Results from the basic numeracy exercise

		*N* = 65 (%)
1	A person taking Drug A has a 1% chance of an allergic reaction. If 1000 people take the drug, how many will have a reaction? (10)	63 (97%)
2	A person taking Drug B has a 1 in a 1000 chance of an allergic reaction. What % of people taking the drug will have a reaction? (0.1%)	54 (83%)
3	The chance of getting a serious viral infection is 0.0005. How many of 10,000 exposed people might get the infection? (5)	38 (58%)
4	Imagine I flip a fair coin 1000 times. How many times will the coin land heads up? (500)	55 (85%)

### Frequency and verbal descriptors

Participants attributed wide numerical ranges to the different verbal descriptors: The term ‘*fairly common’* elicited a range from 10/100 to 90/100; likewise ‘*often’* produced estimates ranged from 1 to 90, ‘*unlikely’* 0.1 to 30 and ‘*very rare’* 0.01 to 10.

### General self-confidence

**Fig. 4:**
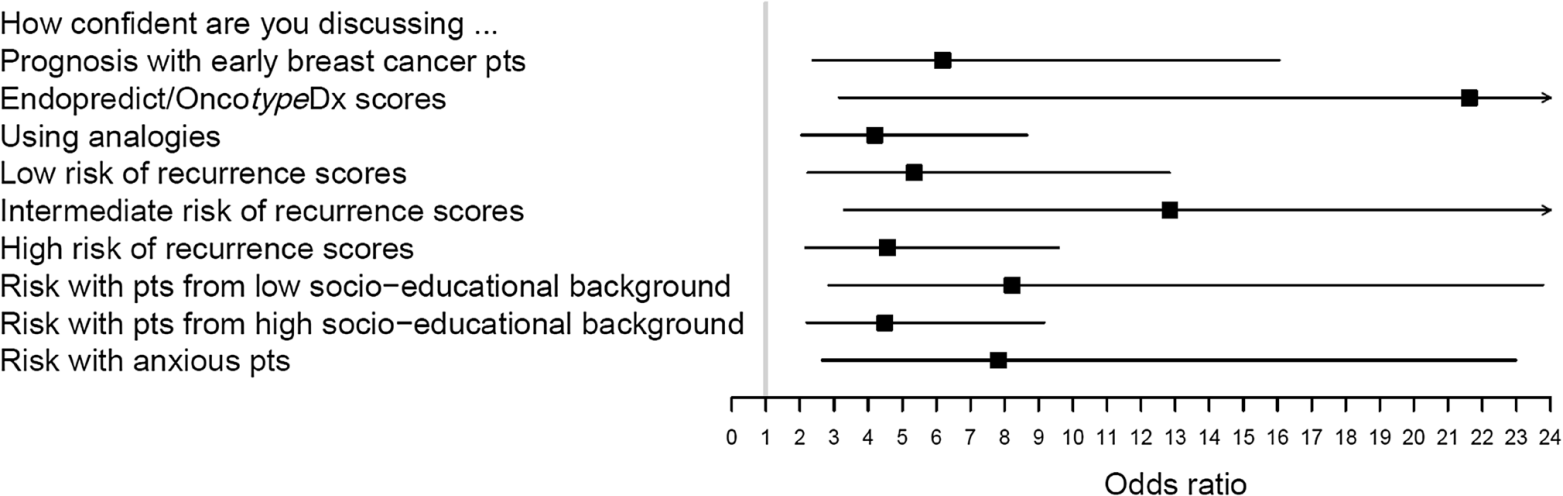
Odds ratios (95% confidence intervals) of improved confidence levels post workshop

Figure 4 shows significant improvements in attendees’ self-confidence for all 9 items—probing prognosis in general, when handling patients with different RSs and those from different socio-educational backgrounds and with varied emotional responses.

Feedback at workshop completion revealed that most attendees found TARGET useful (9.6/10), informative 9.6/10) and enjoyable (9.7/10) and 100% would ‘definitely’ recommend the program to their colleagues.

## Discussion

This intervention was designed specifically to assist HCPs when discussing GEP test scores with patients and implications these have for adjuvant chemotherapy recommendations. There are many generic aspects of the program which are suitable for any HCP tasked with explaining risk to patients.

TARGET was based on successful evidence-based educational packages that contain elements enhancing knowledge acquisition, skills development and self-awareness [17, 18]. Prior research shows that interventions which change both competence and self-confidence/efficacy often transfer successfully into a clinical setting and are enduring [14, 15]. Objective analyses of attendees’ pre- and post-workshop interviews revealed positive behavioural changes in the style and content of discussions and an increase in HCPs’ reported self-confidence.

An important feature of TARGET was enhancement of attendees’ personal awareness about their own numeracy skills and attitudes to uncertainty. Conversations with patients about the rationale and logic for different management policies requires discussion about the potential risks and benefits of adjuvant treatments aimed at reducing recurrence, and the impact that recurrence has on survival. During such discussions many different numbers are provide in dissimilar formats. Numeracy skills of general populations in the US and UK are often poor, and many get confused by numbers with different denominators and experience difficulties with percentages [24]. Studies show that even with decision aids that include graphs, bar charts and icon arrays, some patients have difficulty identifying their own personal risks [25]. It is really important therefore that HCPs communicating risk information can transform numbers into formats appropriate for individual patients in order to truly permit shared/collaborative decision-making. Some TARGET attendees, in common with many highly educated people, had personal numeracy difficulties [26], in particular problems converting percentages into proportions, proportions into percentages and calculating expected frequencies from probabilities.

Using verbal descriptors seems a reasonable way to circumvent numeracy difficulties. Attendees’ responses to the verbal descriptors for common side effect exercise demonstrated widely differing ranges. This revealed the pitfalls if, when discussing risk, numbers are avoided altogether and communicators rely instead on verbal descriptors or frequencies. There is no way of knowing if either their colleagues’ or patients’ interpretations of phrases such as ‘fairly common’ would match their own. The European drug regulatory agency (EMA) has definitions for numbers that should be associated with words relating to side effect frequencies and the Royal College of Anaesthetists has produced pictorial charts assisting understanding about risk, e.g. ‘*rare’* is described as 1:10,000, with a sketch and description of ‘*someone in a small town’* [27].

Ambiguity about management options can ‘leak’ through in obvious and nuanced ways during interviews with patients. Interestingly, those attendees with a high intolerance of uncertainty had lower levels of confidence when discussing intermediate risk of recurrence scores with patients. In clinic settings, these HCPs would be more likely to prescribe chemotherapy especially when faced with an anxious patient, also intolerant of uncertainty, and who had an intermediate or borderline risk score.

One limitation of this work is that unless communication skills programs are made a mandatory part of training, then HCPs who attend workshops maybe a self-selected group, although we saw that the baseline skills of participants ranged widely. TARGET workshops lasted 8 h, split over 2 days but could be adapted for shorter modular-based delivery, lasting 2 h per module. It is unknown whether or not this would produce the same improvements. To assist with wider dissemination, we have produced a facilitator handbook with time-coded transcripts of scenarios enabling facilitators to stop at key points and engage groups in exercises or discussions. There are suggested prompts and comments about the issues illustrated and for less experienced facilitators, examples of structuring teaching sessions. Facilitator training programs are currently being held.

Although TARGET focussed on Onco*type*DX^®^ and EndoPredict^®^ RSs, other available GEP tests share similar communication challenges. Discussing risk and managing the understandable anxiety and uncertainty of women about their need for adjuvant chemotherapy and ultimate decision-making demands a wide repertoire of communication skills that TARGET workshops appeared to enrich.

## Electronic supplementary material

Below is the link to the electronic supplementary material.

Supplementary material 1 (DOCX 41 kb)
