# Supplementary material for: Talking about risk in the context of genomic tests (TARGET): development and evaluation of an educational program for clinicians
Source: Breast Cancer Res Treat. 2019 Jun 14;177(3):641–9. doi: 10.1007/s10549-019-05316-7 (PMC6745030; doi:10.1007/s10549-019-05316-7)
Supplement: Supplementary file 1 — Supplementary material 1 (DOCX 41 kb) [file 10549_2019_5316_MOESM1_ESM.docx]

**Supplementary Tables**

**Table A Odds ratios of improved scores after the course for coder’s objective assessment of HCPs recordings**

| Did the HCP:- | OR | 95% CI | *P* |
| --- | --- | --- | --- |
| say who they were, check patient understood why they’d come to clinic and if needed defined purpose of interview | 3.27 | 1.67, 6.38 | <.001 |
| discuss the surgery, nodal status, ER positivity etc. in reassuring manner | 18.4 | 4.51, 75.14 | <.001 |
| explain adjuvant hormone and radiotherapy was needed to prevent breast cancer recurrence but that benefit of chemotherapy was uncertain | 11.38 | 3.57, 36.29 | <.001 |
| discuss patient’s likely survival with and without adjuvant hormone and chemotherapy, if appropriate/required show survival graphs and check if patient understood them | 1.55 | 1, 2.43 | .043 |
| ask what patient had already been told/knew about purpose of genomic tests and/or explain if needed | 2.98 | 1.38, 6.42 | .001 |
| explain patient’s own genomic test result, risk of recurrence and clarify things if needed, make appropriate use of print outs/graphs to help explain patient’s genomic test risk score | 3.99 | 1.72, 9.25 | <.001 |
| use analogies during the interview to help patient understand numbers/percentages & risk | 2.35 | 1.16, 4.75 | .004 |
| explain the possible benefits of having adjuvant chemotherapy (reduction of risk of recurrence) | 3.99 | 1.82, 8.75 | <.001 |
| explain possible harms of chemotherapy (side-effects, other cancers, burdens of having treatment) | 2.31 | 1.37, 3.92 | <.001 |
| give patient opportunities to ask questions | 1.42 | .68, 2.97 | .352 |
| listen to what patient had to say and respond sensitively to her concerns | 5.2 | 2, 13.54 | <.001 |
| provide sufficient information for patient to make a decision (even if no decision) | 4.24 | 1.9, 9.43 | <.001 |
| involve patient in the decision-making as much as she seemed to require | 2.19 | 1.09, 4.4 | .021 |
| summarise the interview/next steps | 2.87 | 1.48, 5.57 | <.001 |
| ask if patient had any further questions | 1.33 | .99, 1.78 | .048 |
| use clear and understandable, jargon-free language throughout interview, clarifying terminology if needed | 5.29 | 2.27, 12.35 | <.001 |
| the interview was in general well-structured and logically ordered | 12.59 | 3, 52.84 | <.001 |
| Median score | 13.58 | 3.24, 56.83 | <.001 |

**Table B Odds ratios of improved scores after the course for simulated patients’ assessment of communication behaviours**

| How well you believe HCP:- | OR | 95% CI | *P* |
| --- | --- | --- | --- |
| said who they were, checked I understood why I’d come to clinic and if needed defined purpose of interview | 8.44 | 2.63, 27.04 | <.001 |
| discussed the good news about surgery, nodes, fact cancer was ER positive etc. in reassuring manner | 8.98 | 2.85, 28.35 | <.001 |
| explained adjuvant hormone and radiotherapy needed to prevent breast cancer recurrence but that benefit of chemotherapy was uncertain | 1.88 | 1.11, 3.17 | <.001 |
| discussed my likely survival with and without adjuvant hormone and chemotherapy, showed me survival graphs if I wanted to see them and checked if I understood them | 1.63 | .98, 2.69 | <.001 |
| asked what I’d already been told about purpose of genomic tests and/or explained them if needed | 3.79 | 1.96, 7.34 | <.001 |
| explained my genomic test result, risk of recurrence and clarified things if needed, made appropriate use of print outs/graphs if I wanted them to help explain my genomic risk score | 2.87 | 1.48, 5.56 | <.001 |
| used analogies during the interview that helped me understand numbers/percentages and risk | 2.21 | 1.3, 3.75 | <.001 |
| explained the possible benefits of having adjuvant chemotherapy (reduction of risk of recurrence) | 1.27 | .92, 1.76 | <.001 |
| explained possible harms of chemotherapy (side-effects, other cancers, burdens of having treatment) | 2.92 | 1.6, 5.33 | <.001 |
| gave me opportunities to ask questions | 1.94 | 1.12, 3.35 | <.001 |
| listened to what I had to say and responded sensitively to my concerns | 4.82 | 1.92, 12.06 | <.001 |
| provided sufficient information for me to make a decision (even if no decision) | 6.21 | 2.25, 17.14 | <.001 |
| involved me as much as I wanted in the decision-making | 3.61 | 1.65, 7.91 | <.001 |
| summarised the interview/next steps | 2.95 | 1.55, 5.63 | <.001 |
| asked if I had any further questions | 2.36 | 1.35, 4.14 | <.001 |
| used clear and understandable, jargon-free language throughout interview, clarifying terminology when needed* | - | - | .184 |
| the interview was in general well- structured and logically ordered | 4.3 | 1.89, 9.81 | <.001 |
| Median score | 17.80 | 4.5, 70.94 | <.001 |

*Odds ratio not calculable due to a small number of score changes, P value was calculated using Fisher's exact test

**Table C Odds ratios of improved scores after the course for HCP self-assessment of their interviews**

| How well you believe you covered the following:- | OR | 95% CI | *P* |
| --- | --- | --- | --- |
| I introduced myself, checked patient understood why she’d come to clinic and if needed defined purpose of the interview | 6.67 | 1.98, 22.43 | <.001 |
| I discussed the good news about surgery, nodes, fact cancer was ER positive etc. in a reassuring manner | 13.66 | 3.28, 56.88 | <.001 |
| I explained if necessary that adjuvant hormone and radiotherapy was needed to prevent breast cancer recurrence but that the benefit of chemotherapy was uncertain | 21.3 | 2.91, 155.91 | <.001 |
| I discussed patient’s likely survival with and without adjuvant hormone and chemotherapy. If appropriate I showed her survival graphs and checked if she understood them | 1.32 | .75, 2.31 | .325 |
| I asked what she’d already been told about purpose of GEP tests and/or explained if needed | 2.56 | 1.28, 5.12 | .002 |
| I explained patient’s own genomic test result, risk of recurrence and clarified things if needed. I made appropriate use of print outs/graphs to help explain the patient’s genomic test risk score | 3.66 | 1.54, 8.67 | <.001 |
| I used analogies during the interview to help her understand numbers/percentages and risk | 2.8 | 1.32, 5.94 | .003 |
| I explained the possible benefits of having adjuvant chemotherapy (reduction of risk of recurrence) | 5.32 | 1.89, 14.98 | <.001 |
| I explained possible harms of chemotherapy (side-effects, other cancers, burdens of having treatment) | 4.08 | 1.88, 8.85 | <.001 |
| I gave the patient opportunities to ask questions | 2.95 | 1.29, 6.72 | .005 |
| I listened to what the patient had to say and responded sensitively to her concerns | 20 | 2.68, 149.02 | <.001 |
| I provided sufficient information for the patient to make a decision (even if no decision) | 3.29 | 1.26, 8.57 | .006 |
| I involved the patient as much as she seemed to require in the decision-making | 1.79 | .89, 3.59 | .092 |
| I summarised the interview/next steps | 4.61 | 2, 1.66 | <.001 |
| I asked if patient had any further questions | 2.77 | 1.39, 5.55 | <.001 |
| I used clear and understandable, jargon-free language throughout interview, clarifying terminology if needed | 2.43 | 1.01, 5.86 | .038 |
| My interview was in general well-structured and logically ordered | 2 | .75, 5.33 | .153 |
| Median score | 8.14 | 1.90, 34.88 | <.001 |

**Table D Mean (SD) of scores (range 0-3) for coder’s objective assessment of HCPs recordings before and after the course**

|  | Oncologist (n=32) | | Surgeons (n=24) | | Nurses (n=9) | | All (n= 65) | |
| --- | --- | --- | --- | --- | --- | --- | --- | --- |
| Competence item | Before | After | Before | After | Before | After | Before | After |
| introduction & signposting | 2 (0.67) | 2.53 (0.57) | 1.96 (0.69) | 2.25 (0.74) | 1.67 (0.5) | 2.22 (0.83) | 1.94 (0.66) | 2.38 (0.68) |
| discuss the surgery | 2 (0.51) | 2.62 (0.49) | 2.08 (0.78) | 2.88 (0.34) | 1.22 (1.09) | 2.56 (0.53) | 1.92 (0.76) | 2.71 (0.46) |
| explain adjuvant tmts | 1.81 (0.74) | 2.47 (0.57) | 1.92 (0.72) | 2.58 (0.5) | 1.56 (1.13) | 2.11 (1.05) | 1.82 (0.79) | 2.46 (0.64) |
| discuss survival +/-PREDICT | 1.59 (1.07) | 1.91 (1.06) | 1.46 (0.78) | 1.75 (0.99) | 0.11 (0.33) | 0.33 (0.5) | 1.34 (1.02) | 1.63 (1.1) |
| purpose of genomic test | 1.94 (0.5) | 2.06 (0.62) | 1.67 (0.7) | 2.33 (0.48) | 1.22 (0.83) | 1.33 (0.87) | 1.74 (0.67) | 2.06 (0.68) |
| explain RRS +/- chart | 2.31 (0.47) | 2.53 (0.57) | 2 (0.51) | 2.46 (0.59) | 1.44 (0.53) | 2.22 (0.67) | 2.08 (0.57) | 2.46 (0.59) |
| use analogies | 0.16 (0.51) | 0.44 (0.8) | 0.08 (0.41) | 0.54 (1.02) | 0 (0) | 0.22 (0.67) | 0.11 (0.44) | 0.45 (0.87) |
| explain benefit of chemo | 2.06 (0.62) | 2.38 (0.61) | 1.92 (0.58) | 2.38 (0.49) | 1 (0.71) | 1.78 (0.67) | 1.86 (0.7) | 2.29 (0.61) |
| explain harms of chemo | 1.62 (1.01) | 2.12 (1.07) | 1.58 (0.88) | 2.17 (0.7) | 2.11 (0.78) | 2.67 (0.5) | 1.68 (0.94) | 2.22 (0.89) |
| allow opportunities to ask questions | 2.38 (0.49) | 2.41 (0.5) | 2.38 (0.49) | 2.46 (0.51) | 2.44 (0.53) | 2.67 (0.5) | 2.38 (0.49) | 2.46 (0.5) |
| listen to patient's concerns | 2.25 (0.44) | 2.59 (0.56) | 2.38 (0.49) | 2.62 (0.49) | 2.33 (0.5) | 2.78 (0.44) | 2.31 (0.47) | 2.63 (0.52) |
| provide sufficient information | 2.22 (0.55) | 2.66 (0.48) | 2.08 (0.58) | 2.5 (0.66) | 1.89 (0.6) | 2.22 (0.44) | 2.12 (0.57) | 2.54 (0.56) |
| involve patient in the decision-making | 2.12 (0.55) | 2.38 (0.61) | 2.25 (0.44) | 2.38 (0.65) | 2.33 (0.5) | 2.67 (0.5) | 2.2 (0.51) | 2.42 (0.61) |
| summarise the interview/next steps | 2 (0.98) | 2.56 (0.76) | 1.96 (0.75) | 2.46 (0.59) | 2 (1) | 2.33 (0.5) | 1.98 (0.89) | 2.49 (0.66) |
| ask if any further questions | 1.75 (1.19) | 2.06 (1.22) | 1.71 (1.4) | 2.38 (1.13) | 2.11 (1.27) | 2.33 (1) | 1.78 (1.27) | 2.22 (1.15) |
| use clear jargon-free language | 1.97 (0.59) | 2.62 (0.61) | 2.17 (0.7) | 2.42 (0.5) | 2.11 (0.6) | 2.67 (0.5) | 2.06 (0.63) | 2.55 (0.56) |
| interview was well-structured | 1.88 (0.49) | 2.12 (0.55) | 1.92 (0.5) | 2.38 (0.58) | 1.44 (0.53) | 2.11 (0.33) | 1.83 (0.52) | 2.22 (0.54) |

**Table E Mean (SD) of scores (range 0-3) for HCP self-assessment of their interviews before and after the course**

|  | Oncologist (n=32) | | Surgeons (n=24) | | Nurses (n=9) | | All (n= 65) | |
| --- | --- | --- | --- | --- | --- | --- | --- | --- |
| Competence item | Before | After | Before | After | Before | After | Before | After |
| introduction & signposting | 2.16 (0.51) | 2.44 (0.5) | 2.25 (0.44) | 2.5 (0.51) | 2 (0.5) | 2.22 (0.44) | 2.17 (0.49) | 2.43 (0.5) |
| discuss the surgery | 1.94 (0.56) | 2.5 (0.51) | 2.12 (0.54) | 2.42 (0.5) | 1.44 (0.73) | 2.22 (0.67) | 1.94 (0.61) | 2.43 (0.53) |
| explain adjuvant tmts | 1.78 (0.71) | 2.25 (0.51) | 2.08 (0.58) | 2.46 (0.51) | 1.44 (0.53) | 2 (0.71) | 1.85 (0.67) | 2.29 (0.55) |
| discuss survival +/-PREDICT | 1.72 (0.68) | 1.69 (0.74) | 1.67 (0.76) | 1.75 (0.68) | 1.11 (0.78) | 1.78 (0.44) | 1.62 (0.74) | 1.72 (0.67) |
| purpose of genomic test | 1.84 (0.72) | 2.13 (0.76) | 1.67 (0.7) | 2.17 (0.64) | 1.44 (0.73) | 1.67 (0.5) | 1.72 (0.72) | 2.08 (0.7) |
| explain RRS +/- chart | 1.66 (0.6) | 2.03 (0.47) | 1.83 (0.56) | 2 (0.66) | 1.22 (0.44) | 1.67 (0.5) | 1.66 (0.59) | 1.97 (0.56) |
| use analogies | 1.19 (0.83) | 1.41 (0.76) | 1.38 (0.77) | 1.62 (0.71) | 0.56 (0.53) | 1.22 (0.67) | 1.17 (0.81) | 1.46 (0.73) |
| explain benefit of chemo | 1.75 (0.57) | 2.12 (0.55) | 1.92 (0.58) | 2.17 (0.56) | 1.33 (0.71) | 1.78 (0.67) | 1.75 (0.61) | 2.09 (0.58) |
| explain harms of chemo | 1.5 (0.67) | 2.22 (0.75) | 1.88 (0.74) | 2.33 (0.56) | 1.78 (0.83) | 2.11 (0.6) | 1.68 (0.73) | 2.25 (0.66) |
| allow opportunities to ask questions | 2.12 (0.42) | 2.25 (0.57) | 2 (0.78) | 2.46 (0.51) | 2.11 (0.6) | 2.22 (0.83) | 2.08 (0.59) | 2.32 (0.59) |
| listen to patient's concerns | 2 (0.44) | 2.39 (0.56) | 2.21 (0.51) | 2.46 (0.59) | 2.33 (0.5) | 2.44 (0.53) | 2.12 (0.48) | 2.42 (0.56) |
| provide sufficient information | 1.91 (0.53) | 2.16 (0.63) | 1.96 (0.62) | 2.21 (0.51) | 1.89 (0.6) | 1.89 (0.6) | 1.92 (0.57) | 2.14 (0.58) |
| involve patient in the decision-making | 1.97 (0.4) | 2.25 (0.72) | 2 (0.59) | 2.12 (0.74) | 1.89 (0.6) | 1.67 (0.71) | 1.97 (0.5) | 2.12 (0.74) |
| summarise the interview/next steps | 1.69 (0.78) | 2.38 (0.75) | 1.83 (0.56) | 2.08 (0.72) | 1.56 (0.73) | 2 (0.71) | 1.72 (0.7) | 2.22 (0.74) |
| ask if any further questions | 2 (0.72) | 2.34 (0.65) | 1.92 (0.88) | 2.54 (0.59) | 2.33 (0.87) | 2.33 (0.5) | 2.02 (0.8) | 2.42 (0.61) |
| use clear jargon-free language | 1.91 (0.47) | 2.06 (0.5) | 2 (0.51) | 2.17 (0.64) | 2 (0.76) | 2.11 (0.33) | 1.95 (0.52) | 2.11 (0.53) |
| interview was well-structured | 2.04 (0.43) | 2.07 (0.54) | 1.94 (0.54) | 2.11 (0.58) | 1.67 (0.5) | 1.89 (0.33) | 1.95 (0.49) | 2.05 (0.52) |

**Table F Mean (SD) of scores (range 0-3) for simulated patients’ assessment of communication behaviours before and after the course**

|  | Oncologist (n=32) | | Surgeons (n=24) | | Nurses (n=9) | | All (n= 65) | |
| --- | --- | --- | --- | --- | --- | --- | --- | --- |
| Competence item | Before | After | Before | After | Before | After | Before | After |
| introduction & signposting | 2.39 (0.72) | 2.84 (0.45) | 2.33 (0.64) | 2.92 (0.28) | 2.33 (0.87) | 2.67 (0.5) | 2.36 (0.7) | 2.85 (0.4) |
| discuss the surgery | 2.32 (0.65) | 2.88 (0.34) | 2.38 (0.71) | 2.92 (0.28) | 1.67 (1.22) | 2.44 (1.01) | 2.25 (0.8) | 2.83 (0.49) |
| explain adjuvant tmts | 2.41 (0.67) | 2.62 (0.91) | 2.42 (0.72) | 2.71 (0.69) | 1.44 (1.01) | 2.33 (0.71) | 2.28 (0.8) | 2.62 (0.8) |
| discuss survival +/-PREDICT | 2.22 (0.71) | 2.19 (0.9) | 1.92 (1.02) | 2.38 (0.82) | 1.78 (1.09) | 2.56 (0.73) | 2.05 (0.89) | 2.31 (0.85) |
| purpose of genomic test | 2.03 (0.74) | 2.75 (0.57) | 2.12 (0.8) | 2.75 (0.53) | 1.89 (1.27) | 2.44 (1.01) | 2.05 (0.84) | 2.71 (0.63) |
| explain RRS +/- chart | 2.28 (0.85) | 2.62 (0.55) | 2.12 (0.9) | 2.54 (0.59) | 1.67 (0.87) | 2.67 (0.5) | 2.14 (0.88) | 2.6 (0.55) |
| use analogies | 1.88 (0.75) | 2.25 (0.88) | 2.12 (0.95) | 2.38 (0.82) | 1.11 (0.93) | 2.44 (0.73) | 1.86 (0.9) | 2.32 (0.83) |
| explain benefit of chemo | 2.06 (1.01) | 2.22 (1.1) | 1.92 (0.88) | 2.12 (1.19) | 1.44 (1.01) | 2.44 (1.01) | 1.92 (0.97) | 2.22 (1.11) |
| explain harms of chemo | 2.09 (0.78) | 2.81 (0.54) | 2.17 (0.87) | 2.75 (0.53) | 2.44 (0.73) | 2.44 (1.01) | 2.17 (0.8) | 2.74 (0.62) |
| allow opportunities to ask questions | 2.38 (0.83) | 2.62 (0.55) | 2.17 (0.76) | 2.62 (0.58) | 2.78 (0.44) | 3 (0) | 2.35 (0.78) | 2.68 (0.53) |
| listen to patient's concerns | 2.28 (0.81) | 2.78 (0.42) | 2.46 (0.66) | 2.88 (0.34) | 2.78 (0.44) | 3 (0) | 2.42 (0.73) | 2.85 (0.36) |
| provide sufficient information | 2.45 (0.62) | 2.84 (0.37) | 2.33 (0.64) | 2.83 (0.48) | 2.44 (0.73) | 3 (0) | 2.41 (0.64) | 2.86 (0.39) |
| involve patient in the decision-making | 2.22 (0.75) | 2.56 (0.62) | 2.25 (0.68) | 2.71 (0.55) | 2.56 (0.73) | 2.89 (0.33) | 2.28 (0.72) | 2.66 (0.57) |
| summarise the interview/next steps | 2.12 (0.71) | 2.59 (0.67) | 2 (0.78) | 2.5 (0.72) | 2.11 (0.78) | 2.33 (0.71) | 2.08 (0.74) | 2.52 (0.69) |
| ask if any further questions | 2.09 (0.78) | 2.62 (0.71) | 2.12 (0.9) | 2.58 (0.93) | 2.67 (0.5) | 3 (0) | 2.18 (0.81) | 2.66 (0.76) |
| use clear jargon-free language | 2.34 (0.7) | 3 (0) | 2.29 (0.69) | 2.91 (0.29) | 2.22 (0.67) | 2.89 (0.33) | 2.31 (0.68) | 2.95 (0.21) |
| interview was well-structured | 2.36 (0.68) | 2.79 (0.42) | 2.17 (0.71) | 2.72 (0.46) | 2 (0.87) | 2.89 (0.33) | 2.24 (0.72) | 2.78 (0.42) |

**Table G Mean (SD) of scores (range 0-3) for HCPs self-reported confidence levels before and after the course**

|  | Oncologist (n=32) | | Surgeons (n=24) | | Nurses (n=9) | | All (n= 65) | |
| --- | --- | --- | --- | --- | --- | --- | --- | --- |
| How confident are you discussing… | Before | After | Before | After | Before | After | Before | After |
| Prognosis with early breast cancer pts | 7.5 (1.27) | 8.28 (1.02) | 7.58 (1.69) | 8.25 (1.22) | 3.44 (1.51) | 6.22 (2.11) | 6.97 (2.03) | 7.98 (1.45) |
| Endopredict/OncotypeDx scores | 6.97 (1.28) | 8.16 (1.05) | 5.67 (1.61) | 7.79 (1.1) | 2.33 (1.58) | 5.78 (2.11) | 5.85 (2.1) | 7.69 (1.47) |
| Using analogies | 6.38 (1.48) | 7.62 (1.24) | 5.17 (1.76) | 7.54 (1.22) | 2.22 (1.48) | 5.89 (2.09) | 5.35 (2.09) | 7.35 (1.47) |
| Low risk of recurrence scores | 7.56 (1.48) | 8.66 (0.79) | 6.67 (1.37) | 8.5 (0.93) | 2.89 (1.69) | 6.22 (1.72) | 6.58 (2.12) | 8.26 (1.29) |
| Intermediate risk of recurrence scores | 6.12 (1.41) | 7.78 (1.21) | 5.12 (1.3) | 7.33 (1.55) | 2.67 (1.73) | 5.56 (1.88) | 5.28 (1.81) | 7.31 (1.6) |
| High risk of recurrence scores | 7.47 (1.34) | 8.53 (0.88) | 6.54 (1.44) | 8.42 (0.97) | 2.78 (1.48) | 6.22 (2.17) | 6.48 (2.08) | 8.17 (1.39) |
| Risk with pts from low socio-educational background | 6.31 (1.26) | 7.97 (1.15) | 5.67 (1.43) | 7.67 (1.31) | 3.22 (1.99) | 6.33 (1.8) | 5.65 (1.74) | 7.63 (1.4) |
| Risk with pts from high socio-educational background | 6.75 (1.48) | 8 (1.02) | 5.96 (1.71) | 7.96 (1.12) | 3 (1.58) | 6 (1.66) | 5.94 (1.99) | 7.71 (1.33) |
| Risk with anxious pts | 6.41 (1.29) | 7.78 (1.16) | 5.42 (1.59) | 7.46 (1.38) | 2.89 (1.62) | 6 (2.12) | 5.55 (1.85) | 7.42 (1.5) |

**Table H Mean, standard deviation (SD) and range of intolerance of uncertainty scores by HCP’s speciality and sex**

|  | **Oncologist (n=32)** | | | **Surgeons (n=24)** | | | **Nurses (n=9)** | | |
| --- | --- | --- | --- | --- | --- | --- | --- | --- | --- |
|  | Total | PA | IA | Total | PA | IA | Total | PA | IA |
| Mean | 30.03 | 19.06 | 10.97 | 29.88 | 20.46 | 9.42 | 30.11 | 18.78 | 11.33 |
| SD | 8.338 | 5.376 | 3.551 | 3.418 | 2.670 | 2.302 | 9.226 | 5.333 | 4.359 |
| Range | 13-50 | 8-31 | 5-19 | 23-38 | 15-27 | 6-14 | 18-46 | 13-30 | 5-18 |

|  | **Male (n=27)** | | | **Female (n=38)** | | | **All (n=65)** | | |
| --- | --- | --- | --- | --- | --- | --- | --- | --- | --- |
|  | Total | PA | IA | Total | PA | IA | Total | PA | IA |
| **Mean** | 30.26 | 19.81 | 10.44 | 29.79 | 19.34 | 10.45 | 29.98 | 19.54 | 10.45 |
| **SD** | 5.965 | 4.350 | 3.117 | 7.669 | 4.721 | 3.501 | 6.965 | 4.542 | 3.321 |
| **Range** | 20-43 | 12-28 | 6-18 | 13-50 | 8-31 | 5-19 | 13-50 | 8-31 | 5-19 |
